# Identification of long non-coding RNA signatures in triple-negative breast cancer

**DOI:** 10.1186/s12935-018-0598-8

**Published:** 2018-07-17

**Authors:** Tian Tian, Zhouqing Gong, Meng Wang, Ruohan Hao, Shuai Lin, Kang Liu, Feng Guan, Peng Xu, Yujiao Deng, Dingli Song, Na Li, Ying Wu, Zhijun Dai

**Affiliations:** 1 Department of Oncology, Second Affiliated Hospital of Xi’an Jiaotong University, Xi’an, 710004 China; 2 School of Life Science and Technology, Xi’an Jiaotong University, Xi’an, 710049 China; 3 College of Life Science and Technology, Northwest University, Xi’an, 710069 China

**Keywords:** Triple-negative breast cancer, Long non-coding RNA, Bioinformatics

## Abstract

**Background:**

Triple-negative breast cancer (TNBC) is a particular breast cancer subtype with poor prognosis due to its aggressive biological behavior and lack of targets for therapy. This study aimed to explore the expression profile and potential function of lncRNAs in TNBC through bioinformatic methods.

**Methods:**

Two microarrays of TNBC were obtained from the Gene Expression Omnibus database. Differentially expressed lncRNAs and mRNAs were screened out and the expressions of top lncRNAs and overlapping lncRNAs were validated using data from The Cancer Genome Atlas database. The co-expression analysis of lncRNAs and mRNAs was conducted using R software and functional enrichment analysis for was performed by Metascape. Kaplan–Meier Plotter was used for survival analysis.

**Results:**

A total of 1034 dysregulated lncRNAs were found in the two microarrays, and there were 8 overlapped lncRNAs. Among them, 537 lncRNAs were significantly correlated with 451 protein-coding genes (PCGs). The co-expressed PCGs were mainly enriched in terms including cell division, cell cycle, and protein/DNA binding, and were involved in pathways in cancer and other pathways such as PI3K-Akt, MAPK, ErbB and p53 signaling pathways. Hub-genes in the co-expression network were identified, and 7 of them were associated with relapse-free survival of TNBC (*MAGI2*-*AS3*: HR = 0.51; *GGTA1P*: HR = 0.54; *NAP1L2*: HR = 0.59; *CRABP2*: HR = 0.41; *SYNPO2*: HR = 0.50; *MKI67*: HR = 2.23; *COL4A6*: HR = 1.91; all *P *< 0.05).

**Conclusions:**

Numerous lncRNAs were dysregulated in TNBC, and many of them are possibly involved in cancer biology. Several of these lncRNAs were associated with of TNBC prognosis, which can be promising biomarkers.

**Electronic supplementary material:**

The online version of this article (10.1186/s12935-018-0598-8) contains supplementary material, which is available to authorized users.

## Background

Breast cancer (BC) is the most common type of cancer and the leading cause of cancer death among women all over the world. [1]. Triple-negative breast cancer (TNBC) is a particular subtype of breast cancer, characterized by poor prognosis because of its aggressive biological behavior and lack of molecular targets for therapy [2]. It is defined by the absence of estrogen receptor (ER) and progesterone receptor (PR) expression and without amplification of human epidermal growth factor receptor 2 (HER2) [3]. The treatment methods for TNBC are very limited owing to the lack of decisive therapeutic targets. Hence, it is necessary to explore new targeted approaches and make efforts to improve the outcomes of TNBC.

In recent years, long non-coding RNAs (lncRNAs) have drawn an increased attention because of their functions in the human diseases including cancers. LncRNAs are a class of RNA transcripts with a length of > 200 nucleotides that do not encode proteins. They are involved in diverse biological processes such as cell proliferation, differentiation, chromosome remodeling, epigenetic modulation, transcriptional and posttranscriptional modifications [4]. Studies have revealed that lncRNAs play an important role in cancer biology and the expression level or mutation of specific lncRNA genes are implicated in the development and progression of cancer. Moreover, a large number of lncRNAs are deregulated in multiple tumors including breast cancer, making them possibly as diagnostic and prognostic biomarkers or as potential therapeutic targets for cancer [5, 6]. Several lncRNAs have been reported to regulate TNBC progression. For instance, lncRNA *LINP1* is overexpressed and enhances double-strand DNA break repair in TNBC. Blocking *LINP1* increases sensitivity of BC cell response to radiotherapy [7]. *LINK*-*A* facilitates the activation of BRK kinase, thus activates normoxic HIF1α signaling in TNBC, promoting breast cancer glycolysis reprogramming and tumorigenesis [8]. With the development of RNA sequencing and genomic technologies as well as computational techniques, more and more lncRNAs have been discovered. However, the studies about lncRNAs and TNBC are very few by far, and the expression profile, functions and mechanisms of lncRNAs in TNBC remains to be extensively explored [9, 10]. Thus, we mined and analyzed data from several databases, hoping to highlight signatures of lncRNAs in TNBC and provide foundation for further studies.

## Methods

### Acquisition and analysis of microarray data

Two lncRNA microarray datasets (GSE60689 and GSE64790) of TNBC were obtained from the Gene Expression Omnibus (GEO) database (https://www.ncbi.nlm.nih.gov/geo/). Raw data were reprocessed by the online tool GEO2R. The annotation of lncRNAs was accessed directly from additional files of the microarrays or query from the LNCipedia database (http://www.lncipedia.org, version 5.0). If a lncRNA has an official gene symbol according to HGNC, that symbol was used as the name of lncRNA. Otherwise, the accession number or gene ID was used. We then screen out significant differentially expressed lncRNAs for further analysis with the criteria of |lgFC| ≥ 2.0 and P value ≤ 0.05. The overlapping lncRNAs were identified through an online tool for Venn diagram (http://bioinformatics.psb.ugent.be/webtools/Venn/). The software Morpheus (https://software.broadinstitute.org/morpheus/) were used to draw heatmap.

### Validation of lncRNA genes expression

The breast invasive carcinoma (BRCA) RNAseq dataset from The Cancer Genome Atlas (TCGA) database were used to validate the expression profile of the top 10 lncRNAs and the overlapped lncRNAs in two microarrays. Data were downloaded through the Atlas of ncRNA in cancer (TANRIC) database (http://ibl.mdanderson.org/tanric/_design/basic/index.html) [11]. The lncRNA expression level was quantified using log_2_RPKM value. And t or t’ test were used to examine the difference between TNBC and normal groups.

### Co-expression analysis of lncRNAs with mRNAs

The microarray GSE64790 also investigated the expression profile of mRNAs in TNBC. Therefore, we selected differentially expressed mRNAs with the same criteria for lncRNAs and then conducted co-expression analysis for the differentially expressed lncRNAs and mRNAs using R software. Pearson’s correlation coefficients between the lncRNA genes and mRNA genes were calculated using the expression matrix. P < 0.01 was the cut-off value to define significant correlations. The co-expression network was constructed by the software Cytoscape (version 3.5.1), and hub-genes in the network were selected according to their rank by degree [12].

### Functional enrichment analysis for DEGs

We mined BC-associated genes reported by literature through PALM-IST (http://www.hpppi.iicb.res.in/ctm/index.html). The overlapping genes in BC-associated gene set and co-expressed differentially expressed gene (DEG) set were screened out for functional enrichment analysis, performing by Metascape (http://metascape.org) [13]. Gene Ontology (GO) terms for the biological process (BP), cellular component (CC) and molecular function (MF) categories as well as Kyoto Encyclopedia of Genes and Genomes (KEGG) pathways were enriched. Only terms with P value < 0.01 and the number of enriched genes ≥ 3 were concerned as significant. All the resultant terms were then grouped into clusters based on their similarities. The most enriched term within a cluster was chosen as the one to represent the cluster.

### DEG-based survival analysis

The online survival analysis tool Kaplan–Meier Plotter (http://kmplot.com/analysis/) was used to assess the prognostic value of these significant DEGs in our analysis in TNBC. Patients were divided into high and low expression groups according to the median expression level of the corresponding gene. The log-rank test was used to examine the significance of difference between two groups and HR was calculated to evaluate the association of gene expression with survival [14].

## Results

### Dysregulated lncRNAs in TNBC

**Fig. 1:**
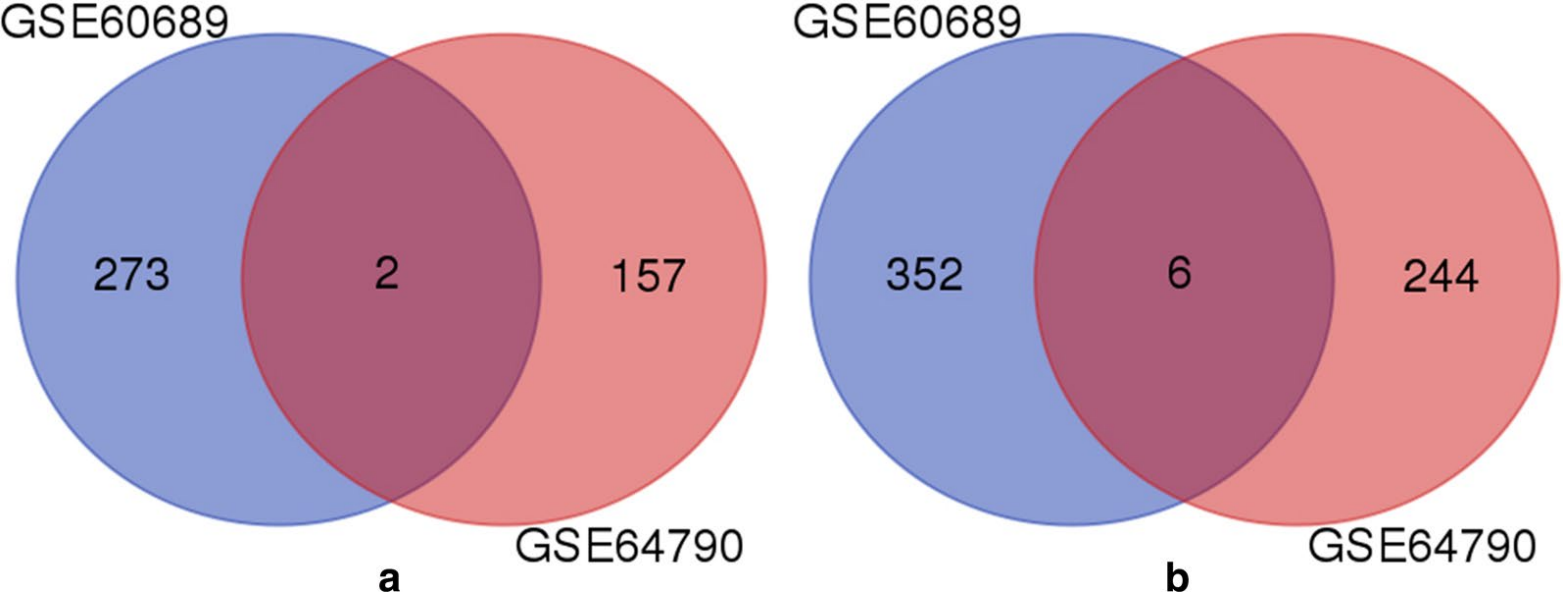
Venn diagram for overlapping lncRNAs in the two microarray datasets: **a** two lncRNAs were up-regulated in both microarrays; **b** six lncRNAs were down-regulated in both microarrays

**Fig. 2:**
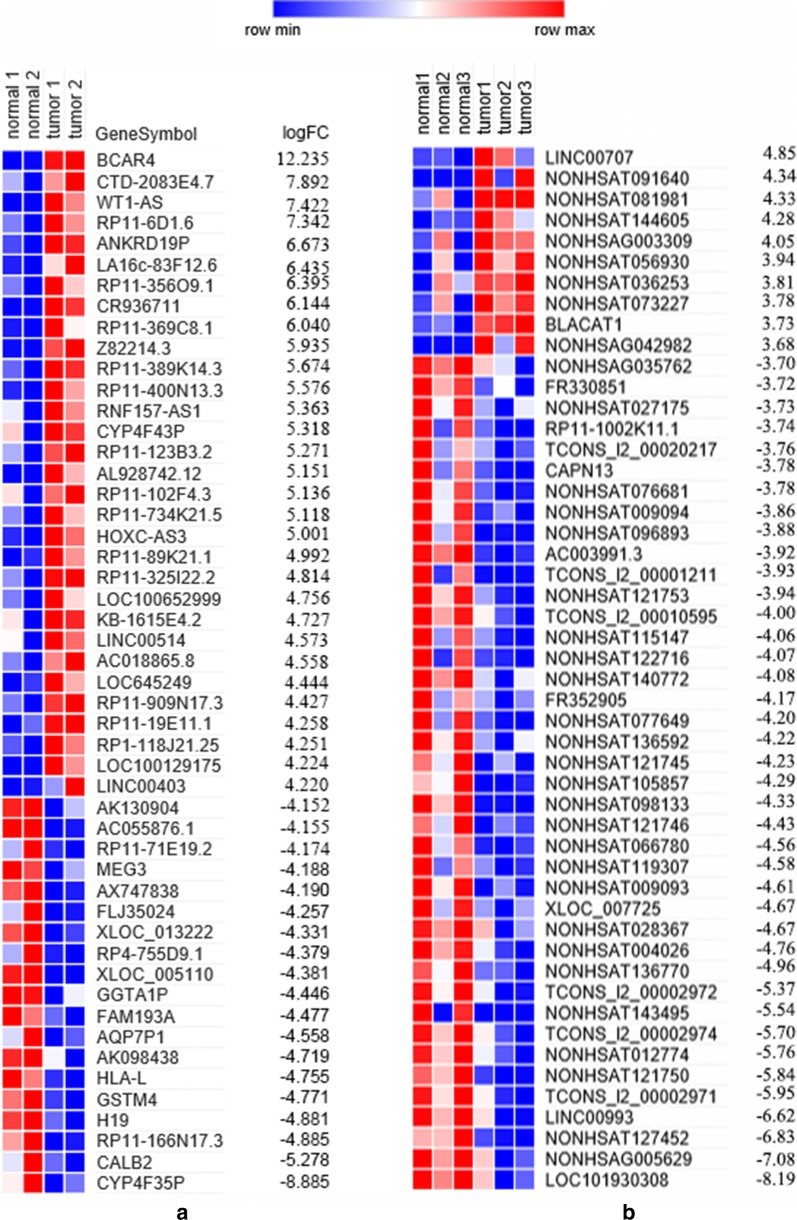
Heatmap for the top 50 differentially expressed lncRNAs in: **a** microarray GSE60689; **b** microarray GSE64790

**Fig. 3:**
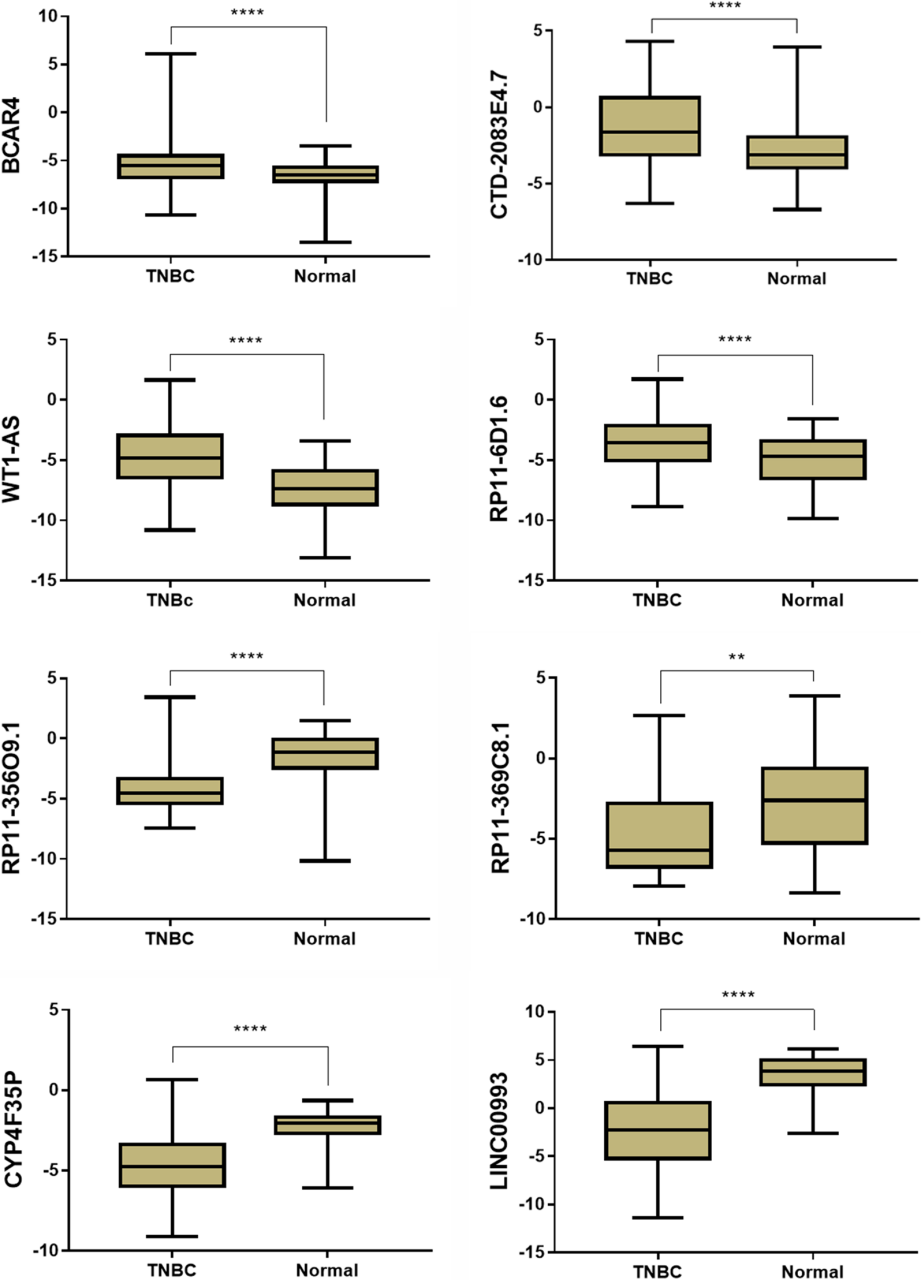
The expression of eight lncRNAs in TNBC. These lncRNAs were among the top 10 differentially expressed lncRNAs in the two microarrays, and their expressions were validated with data from BRCA cohort of TCGA

**Fig. 4:**
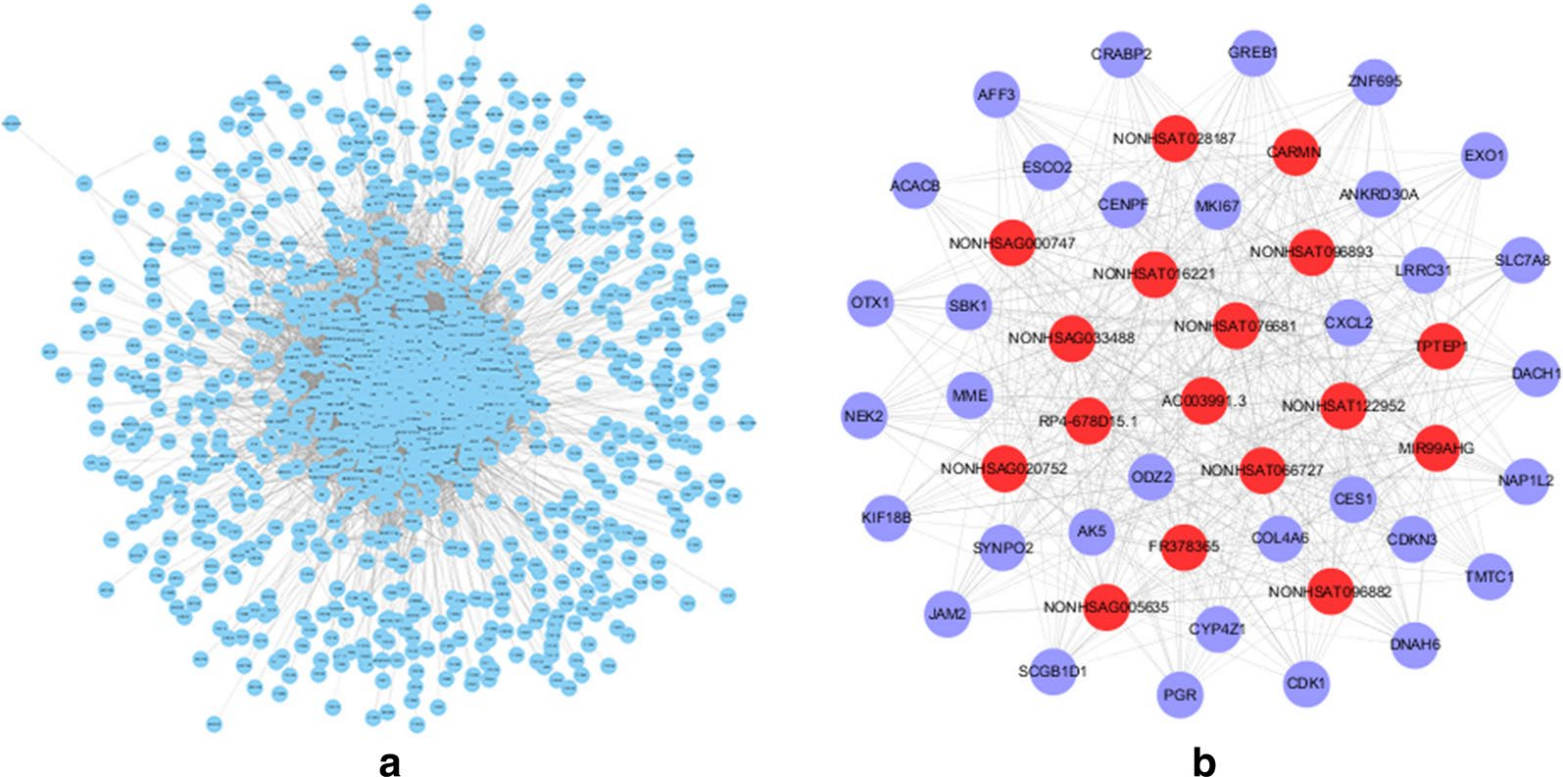
Co-expression network of: **a** all the significantly correlated DEGs (P < 0.01); **b** top 50 hub-genes among the whole network. The red and blue nodes denote lncRNA genes and protein-coding genes respectively

Through analysis of microarray data, 432 up-regulated lncRNAs and 602 down-regulated lncRNAs were identified according to our criteria (Additional file 1: Table S1). There are 8 overlapped lncRNAs (2 up-regulated and 6 down-regulated) in both microarrays (Fig. 1 and Table 1). The 50 top differentially expressed lncRNAs in the two microarrays were shown in Fig. 2. Of the top 10 lncRNAs in the two microarrays, 8 were found in TANRIC database. Their expressions were validated in 119 TNBC samples and 105 normal controls with data from BRCA RNAseq dataset of TCGA. As shown in Figs. 3, 4 lncRNAs were increased while the other 4 lncRNAs were decreased in TNBC compared with normal tissues (*P *< 0.01). The expressions of all these lncRNAs were consistent with the results of microarrays except two (RP11-356O9.1 and RP11-369C8.1).

**Table 1:** Differentially expressed lncRNAs in both microarrays

Gene symbol	Full name	Location	Expression
LINC00511	Long intergenic non-protein coding RNA 511	Chr 17	Up
MNX1-AS1	MNX1 antisense RNA 1	Chr 7	Up
MEG3	Maternally expressed 3	Chr 14	Down
MIR22HG	MIR22 host gene	Chr 17	Down
RP11-305O6.3	N/A	Chr 12	Down
LINC01091	Long intergenic non-protein coding RNA 1091	Chr 4	Down
XLOC_009135	N/A	Chr 11	Down
MAGI2-AS3	MAGI2 antisense RNA 3	Chr 7	Down

### Co-expression network and hub-genes

The co-expression analysis showed that there are 537 nonco-lncRNAs and 451 protein-coding genes (PCGs) whose expressions are significantly correlated. A co-expression network of these DEGs was constructed based on their correlation coefficients. The network is very large comprising 1259 nodes and approximately 40 thousand edges, including 25,203 positive connections and 14,449 negative connections (Fig. 4a). The 50 top hub-genes were selected out and visualized (Fig. 4b). Among these hub-genes, 17 were lncRNA genes and 33 were PCGs.

### Functional characterization of DEGs

**Fig. 5:**
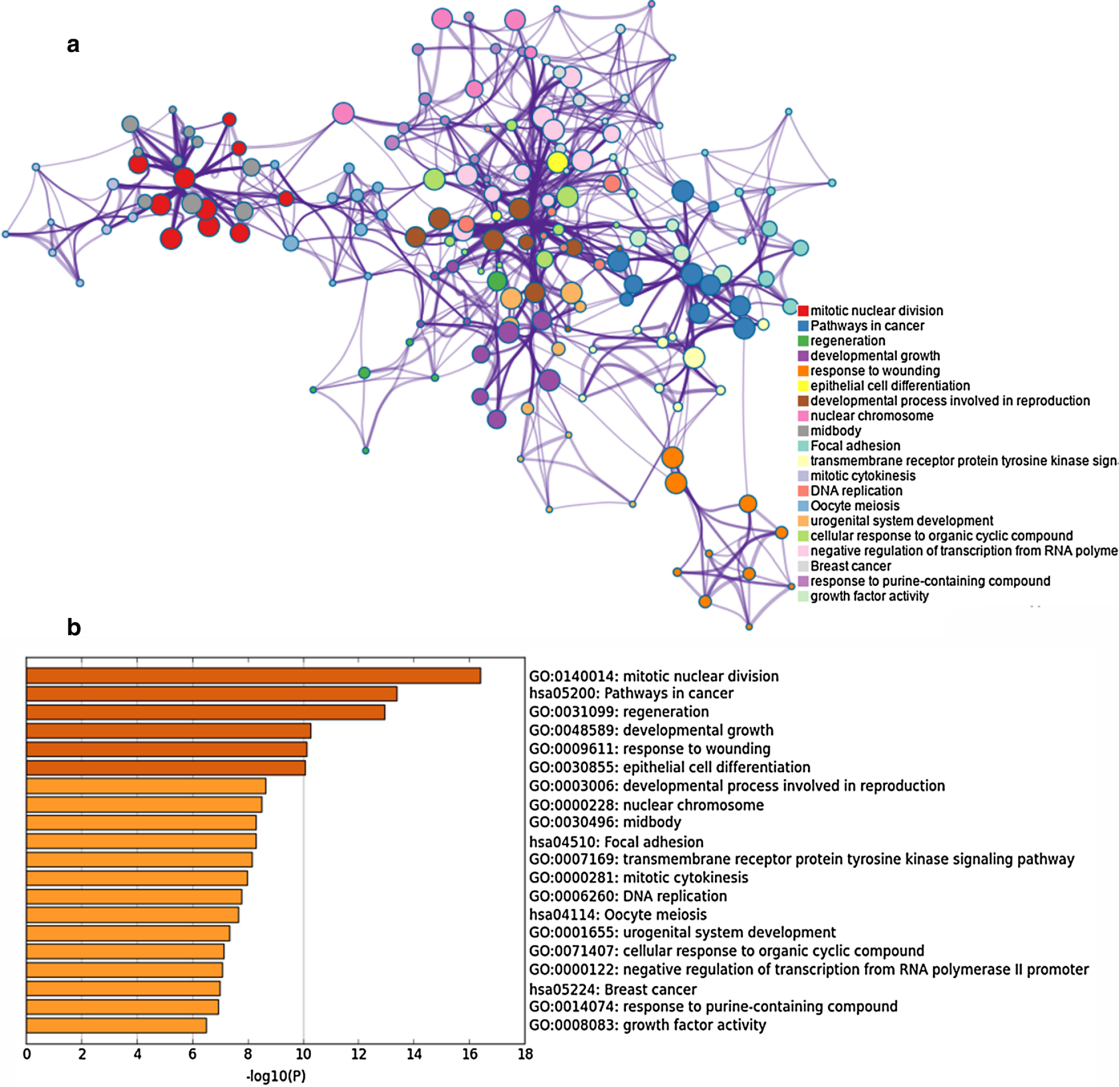
Functional enrichment results: **a** network of top 20 clusters of enriched terms. Each node represents one enriched term colored by cluster ID, nodes share the same cluster are typically close to each other. Terms with Kappa similarity above 0.3 were connected. The thicker edge displayed, the higher the similarity is. The term with the best *P* value was selected to represent each cluster; **b** heatmap of top 20 clusters, colored by P-values. The smaller the P-value is the deeper the color is

In total, 1037 terms were enriched, including 826 BP terms, 62 CC terms, 89 MF terms and 60 KEGG pathways (Additional file 2: Table S2). The DEGs mainly involve in biological process such as cell division, chromosome segregation, and cell cycle, and have molecular function such as protein and DNA binding, protein kinase activity, receptor ligand or regulator activity. Most of enriched pathways are cancer-related. A total of 25 DEGs are involved in pathways in cancer, which is the most enriched one. Other pathways include focal adhesion, breast cancer, cell cycle as well as PI3K-Akt, MAPK, ErbB and p53 signaling pathway, etc. The top 20 clusters of significantly enriched terms are shown in Fig. 5.

### Prognostic value of hub-genes in TNBC

**Fig. 6:**
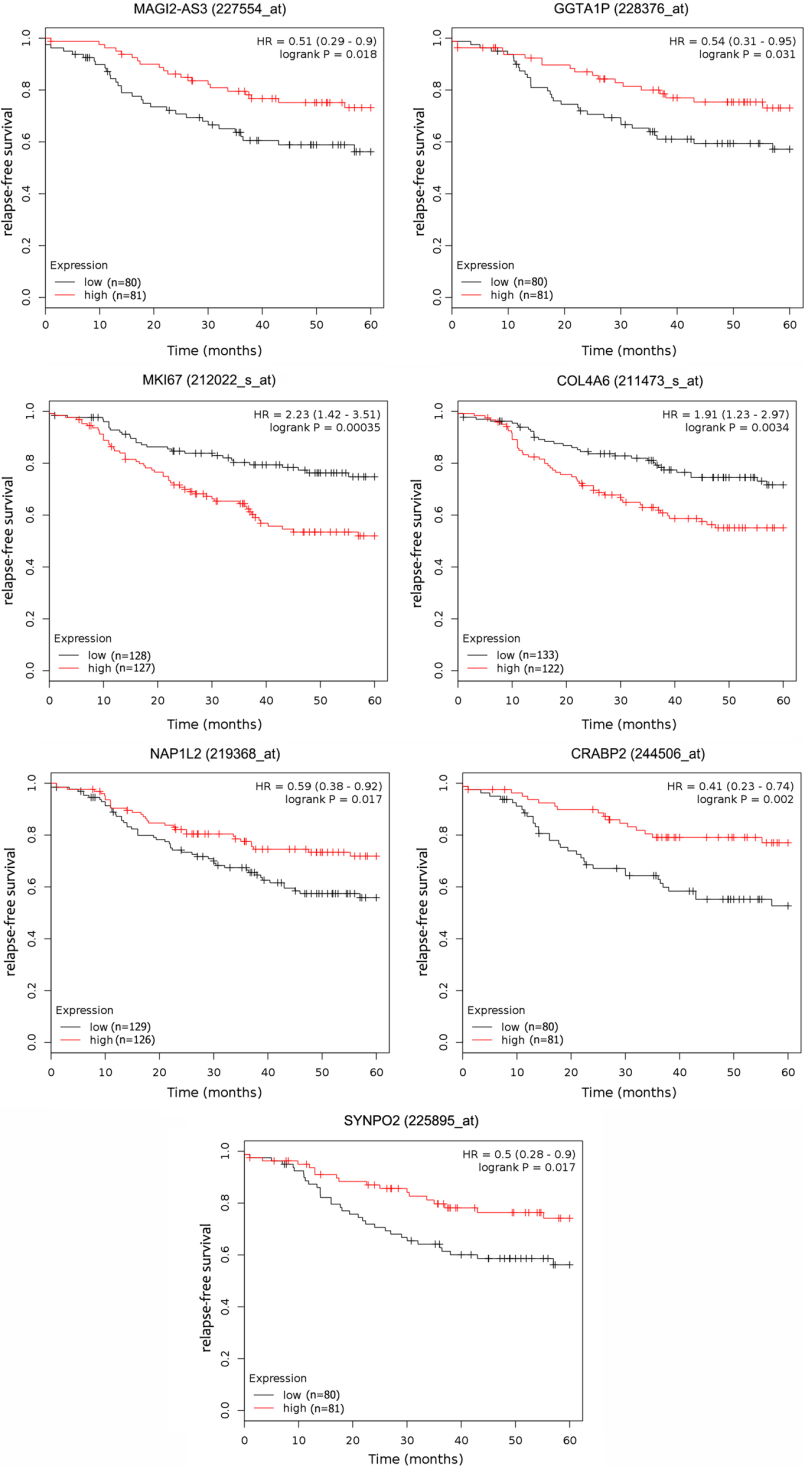
The association between the expression of seven DEGs and the survival of TNBC patients

In order to explore the prognostic values of the significantly dysregulated lncRNAs, we analyzed the associations of the expression level of 50 top hub-genes in co-expression network with the survival of TNBC patients. Seven DEGs (2 lncRNA genes and 5 PCGs) were found to associate with relapse-free survival (RFS) of TNBC (*P *< 0.05, Fig. 6). Patients with elevated level of *MAGI2*-*AS3* and *GGTA1P* tend to have a better relapse-free survival (HR = 0.51 and 0.54 respectively). In addition, high expression of *NAP1L2*, *CRABP2* and *SYNPO2* is beneficial for RFS (HR = 0.59, 0.41 and 0.50 respectively) of TNBC patients, whereas increased level of *MKI67* and *COL4A6* is a risk factor for RFS (HR = 2.23 and 1.91 respectively).

## Discussion

Among all the breast cancer subtypes, TNBCs account for approximately 15% to 20% of all diagnosed breast cancer cases and are more prevalent in younger women (age < 40 years) [15]. TNBC is a complex and heterogeneous disease and the outcomes of patients are relatively worse than those of other subtypes. Only 30–45% of TNBC patients can achieve a pathological complete response (pCR) and survival rates similar to other types of breast cancer [16]. The poor prognosis of TNBC is mainly due to the lack of effective targets for treatment. Therefore, it is crucial to find new therapeutic targets for the improvement of TNBC prognosis.

LncRNAs play an important role in carcinogenesis. Many lncRNAs are dysregulated in tumors, and they are promising diagnostic biomarkers and potential therapeutic targets for cancers [17–19]. In this study, we identified a number of TNBC-associated lncRNA genes through bioinformatic methods. Most of them are novel lncRNAs, many of which even do not have an official name. All of the overlapped lncRNAs in both microarrays have not been studied in BC except MEG3. So, they are good targets for future research. There are also some lncRNAs which have been extensively studied previously. For example, the most dysregulated lncRNA gene, *BCAR4*, has been found to be overexpressed in breast tumor tissue in previous studies and was associated with poor survival of breast cancer patients [20, 21]. Furthermore, it has been proved that *BCAR4* can promote breast cancer cell migration and invasion through noncanonical hedgehog signaling pathway [21]. *MEG3* is a tumor suppressor lncRNA gene, its expression is decreased in multiple tumors including lung cancer, gastric cancer, hepatocellular carcinoma, glioma etc. [22]. In breast cancer, it can inhibit cell proliferation, invasion and angiogenesis by sponging microRNAs and regulating signaling transduction such as AKT and TGF-β pathway [23, 24]. *H19* is also one of the major lncRNA genes in cancer, but it has long been a controversy whether it is oncogenic or tumor-suppressive. *H19* plays a role in tumor initiation and progression, the mechanisms, however, vary among cancer types [25, 26]. In breast cancer, *H19* involves in tumor growth and metastasis through interaction with protein and microRNAs [27]. The mechanisms of lncRNA regulation in TNBC have not been clarified by far. Previous studies have shown that they can be regulated by some important signaling pathways. For example, *LINP1* expression is activated by the EGF signaling and repressed by the p53 pathway in TNBC [7]. The expression level of lncRNAs can also be altered by epigenetic modification. For example, the promoter-associated CpG island of LOC554202 was hypermethylated, thus leading to the down-regulation of LOC554202 in TNBC cells [28]. In addition, lncRNA’s expression can be regulated by its biodegradation rate or transcription rate [29].

There were some similar studies published previously [30–32]. But these studies only mined data from one microarray without making in-depth analysis. Our study comprehensively analyzed two datasets and made further analysis. We also identified numerous abnormally expressed PCGs in TNBC. And by establishing gene co-expression network, we found the PCGs whose expression profiles are correlated with that of lncRNA genes. Many of these PCGs were enriched in biological processes and pathways which are important for tumorigenesis and cancer progress. The 50 top genes ranked by degree in the network were selected as hub-genes, among which, low expression of three genes (*NAP1L2*, *CRABP2* and *SYNPO2*) while high expression of two genes (*MKI67* and *COL4A6*) was associated with poor RFS of TNBC patients. The products of hub PCGs mainly function as protein binding molecule and were involved in important biological processes and signaling pathways in cancer (*CD*K1, *MKI67*, *CENPF*, *COL4A6*, *DACH1*, etc.). As they are highly correlated with lncRNA genes, they may be the targets through which the TNBC-associated lncRNAs can influence the onset and progress of TNBC.

## Conclusions

In summary, numerous lncRNA were dysregulated in TNBC and many of them are possibly involved in cancer development. The specific function of these lncRNAs needs further exploration. Nevertheless, our study illuminates a comprehensive understanding of lncRNA signatures in TNBC and suggests its important role. These dysregulated lncRNAs can be promising biomarkers for diagnosis or prognosis and may be potential targets for therapy. We hope these findings can draw more attention to lncRNAs in TNBC research and provide orientations for future studies.

## Additional files

**Additional file 1: Table S1.** Differentially expressed lncRNAs in the two microarrays.

**Additional file 2: Table S2.** Functional enrichment terms of DEGs.

## Abbreviations

TNBC: triple-negative breast cancer
lncRNA: long non-coding RNA
DEG: differentially expressed gene
PCG: protein-coding gene
GEO: Gene Expression Omnibus
TCGA: The Cancer Genome Atlas
ER: estrogen receptor
PR: progesterone receptor
HER2: human epidermal growth factor receptor 2
RFS: relapse-free survival
